# Silicon germanium photo-blocking layers for a-IGZO based industrial display

**DOI:** 10.1038/s41598-018-35222-9

**Published:** 2018-12-03

**Authors:** Su Hyoung Kang, Sangmin Kang, Seong Chae Park, Jong Bo Park, Youngjin Jung, Byung Hee Hong

**Affiliations:** 1 Department of Chemistry, College of Natural Science, Seoul National University, Seoul, 08826 Korea; 2 Department of Electrical and Computer Engineering, University of Illinois at Urbana-Champaign, Urbana, Illinois 61801 USA; 3 Graphene Research Center, Advanced Institute of Convergence Technology, Seoul National University, Suwon, 16229 Korea

## Abstract

Amorphous indium- gallium-zinc oxide (a-IGZO) has been intensively studied for the application to active matrix flat-panel display because of its superior electrical and optical properties. However, the characteristics of a-IGZO were found to be very sensitive to external circumstance such as light illumination, which dramatically degrades the device performance and stability practically required for display applications. Here, we suggest the use for silicon-germanium (Si-Ge) films grown plasma-enhanced chemical vapour deposition (PECVD) as photo-blocking layers in the a-IGZO thin film transistors (TFTs). The charge mobility and threshold voltage (V_th_) of the TFTs depend on the thickness of the Si-Ge films and dielectric buffer layers (SiN_X_), which were carefully optimized to be ~200 nm and ~300 nm, respectively. As a result, even after 1,000 s illumination time, the V_th_ and electron mobility of the TFTs remain unchanged, which was enabled by the photo-blocking effect of the Si-Ge layers for a-IGZO films. Considering the simple fabrication process by PECVD with outstanding scalability, we expect that this method can be widely applied to TFT devices that are sensitive to light illumination.

## Introduction

As a panel size has been changed to large scale screen size in the active-matrix flat panel displays, many researchers have investigated in transparent metal oxide semiconductors instead of amorphous silicon because of their high mobility and low synthesis temperature (~250 °C)^1–3^. In particular, amorphous indium-gallium-zinc oxide (a-IGZO) have been suggested for a future active material, which shows superior charge mobility and threshold gate swing (~0.083 V/decade)^4^. In addition, a-IGZO based thin film transistors (TFTs) have attracted more attention due to its transparent optical properties and convenient synthesis methods at low temperature with reasonable cost^5^.

In display industry, the top gate TFT method has been widely used for the process architecture to create liquid crystal display (LCD) and organic light emitting diode (OLED)^6,7^. Top gate method has an advantage to increase an aspect ratio compared to the bottom gate method^8,9^. However, in case of top gate method, it could not avoid that a-IGZO reacts with the light leakage originated from backlight in LCD display^10,11^. IGZO has been known that the optical bandgap is E_g_ = 3.2 eV, which indicates that it could be activated on the condition of UV wave length less than 400 nm^12,13^. Through the inevitable reaction with UV range in light leakage, the electrical properties and reliability tests for long term panel displays show considerable degradation of the performance^14^. The attributions to degradation of the electrical properties are trapping of holes created by photo-generation and instability in the interface between a-IGZO and dielectric materials^15^.

Recently, a few research groups have focused on metal based photo blocking layers for a-IGZO films^16–18^. Although the metal based photo blocking layers prevent the reaction of a-IGZO films from the backlight, the performance of the a-IGZO TFTs is decreased due to the increased cap size between metal and IGZO. In addition, the high power consumption is also needed to increase the brightness of a backlight for the purpose of keeping the brightness of screen^19,20^. Therefore, the demand for the photo blocking layers composed of new materials different from conventional metal layers is continuously raised in display industry.

In this study, we suggest the silicon-germanium (Si-Ge) films as the photo-blocking layers in a-IGZO based TFTs. According to preceding research, ultraviolet (UV) absorbance is increased in the Si-Ge alloy which have more Ge ratio than Si ratio^21^. However, the difference of UV absorbance depending on components ratio is not enough to satisfy the absorbance condition for a photo blocking layer. In order to find better results, we controlled the thickness of the Si-Ge films which have fixed component ratio (Si:Ge = 1:1). It was found that TFTs with Si-Ge photo blocking layers preserved the initial value including mobility and threshold voltage (V_th_) without any oxidation or chemical damages under the light illumination.

## Results

**Figure 1:**
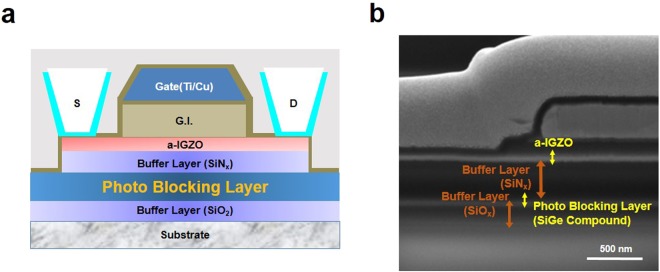
Entire structure of a-IGZO based display passivated by photo blocking layer. (**a**) Schematic illustration of the Si-Ge photo blocking layer for industrial a-IGZO TFT degradation. (**b**) Side view SEM image of a-IGZO TFT passivated by the Si-Ge films.

The Si-Ge films were synthesized on glass substrates coated by silicon oxide by plasma-enhanced chemical vapor deposition (PECVD). Without the silicon oxide, it is difficult to directly grow the Si-Ge films on the glass substrate. Fig. 1a shows a schematic illustration of the structure of a-IGZO TFTs passivated by the Si-Ge photo-blocking layers with the top gate and electrode contact. Silicon Nitride buffer layers were deposited on the Si-Ge layers for dielectric materials. The synthesis method is more explained in following experimental synthesis parts. Fig. 1b is the side image of the structure of a-IGZO TFTs measured by scanning electron microscopy (SEM). We confirmed that uniform Si-Ge films were placed among the two oxide based dielectric layers without any externally damage to overall structure.

**Figure 2:**
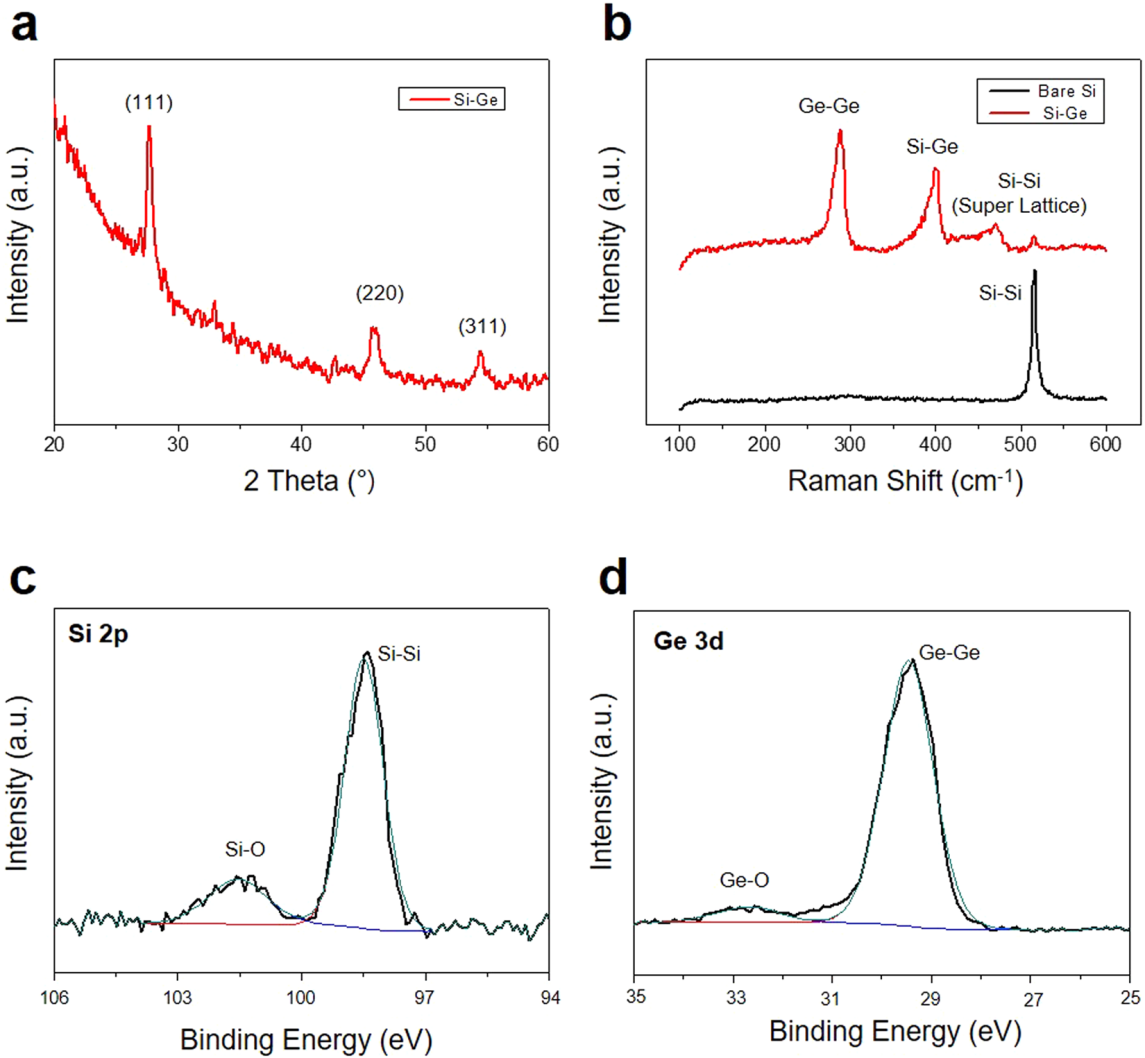
Material characterization of the Si-Ge photo blocking layers. (**a**) XRD patterns of the Si-Ge films. (**b**) Raman spectra of the Si-Ge photo blocking layers compared with a bulk Si. XPS spectra of the Si-Ge films showing signals of (**c**) Si 2p and (**d**) Ge 3d.

**Figure 3:**
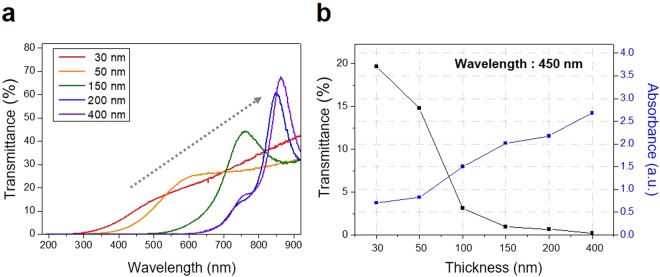
UV-Vis spectrum analysis of the Si-Ge films. (**a**) UV-Vis spectrum according to the thickness of the Si-Ge films. (**b**) UV absorbance of the Si-Ge films at 450 nm.

The crystallinity of the Si-Ge films synthesized by PECVD was investigated by X-ray diffraction measurements (XRD). As shown in Fig. 2a, the XRD patterns of the Si-Ge films exhibit conventional cubic phase similar to that of bulk silicon (JCPDS No. 27–1402). Reflections of the Ge phase are imperceptible in the XRD patterns, suggesting that Ge exists as a solid solution^22^. The formation of the Si-Ge bonding is more clearly explained by Raman spectrum as shown in Fig. 2b. The Raman peaks at 510, 405 and 295 cm^−1^ from Si-Si, Si-Ge and Ge-Ge bonding, respectively, are observed. In particular, the additional Raman peaks between 420 and 470 cm^−1^, which are the vibrational modes of Si super lattice, are measured. These vibrational modes which are composed of localized Si-Si optical modes whose frequencies are lowered because of the larger mass of adjacent Ge atoms^23^. X-ray photoelectron spectroscopy (XPS) also demonstrates chemical composition and surface oxidation of the Si-Ge films. The Si 2p and the Ge 3d peaks obtained from XPS could be ascribed to elemental Si and Ge, as shown in Fig. 2c,d. Owing to slight surface oxidation, weak peaks related oxygen are also detected around 101.5 eV at Fig. 2c and 33 eV at Fig. 3d, respectively. These characterizations summarize that the elemental phases are originated from Si and Ge elements and their bonding.

Figure 3a shows the ultra violet – visible (UV-Vis) spectrum according to the thickness of the Si-Ge compounds. The UV absorbance peak was gradually shifted in which direction increasing the thickness of Si-Ge layers according above the equation. In particular, we confirmed the UV absorbance at 450 nm more detail, which the a-IGZO react with actively (Fig. 3b)^24^. Photo blocking effect is also proportional to the thickness like as other wavelength. Therefore, it is clear that the higher thickness of Si-Ge compounds has good blocking effects, thereby offering a possibility for superior photo blocking layers in a-IGZO based displays.

**Figure 4:**
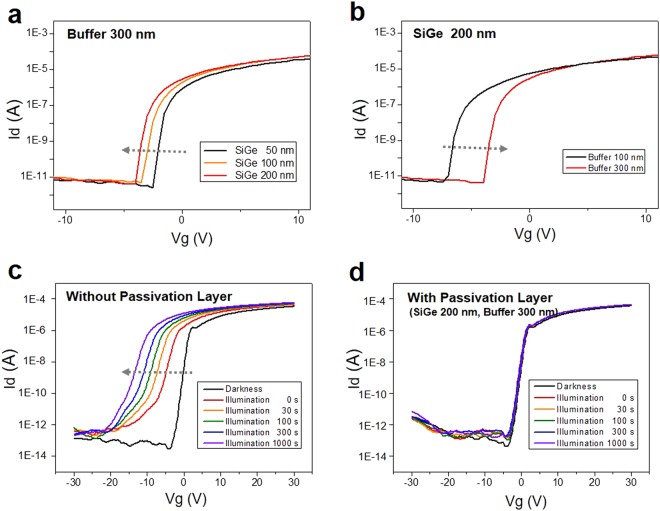
Electrical characteristics of the a-IGZO TFTs. (**a**) Transfer Characteristics depending on the thickness of the Si-Ge films at dielectric buffer layers 300 nm. (**b**) Transfer Characteristics depending on the thickness of the dielectric buffer layers at the Si-Ge films 200 nm. Representative electrical properties of a-IGZO TFTs (**c**) without and (**d**) with the Si-Ge based photo blocking layers.

Although the higher thickness of the Si-Ge films has outstanding UV absorbance, the electrical superiority is inversely proportional to the thickness. Figure 4a shows the threshold voltage (V_th_) is negatively shifted depending on the thickness of the Si-Ge films at dielectric buffer layers 300 nm. (Supplementary Fig. S1) The more charge carrier density, so called free electrons, are accumulated in the Si-Ge films as the thickness of the Si-Ge films is increased. The accumulated electrons make the Si-Ge films more conductive, thus making it a kind of capacitance with a-IGZO. Eventually this accumulated charge carriers cause the a-IGZO channel to shut off while generating tunnelling effects to the Si-Ge layers. Therefore, we optimize the thickness of Si-Ge compounds without any tunnelling effects. In addition, the thickness of the silicon nitride buffer layers could also give rise to the performance of the a-IGZO TFTs, as shown in Fig. 4. (Supplementary Fig. S2) The more deposition of dielectric materials results in the improved efficiency of TFTs due to the decrease in capacitance^25,26^. However, too high thickness of buffer layers lead to surface instability, which makes post processes to fabricate the TFTs very difficult. Hence, we selected the heights of the Si-Ge films and the silicon nitride buffer layers to 300 nm and 200 nm, respectively for achieving maximum TFT efficiency. Figure 4c shows the evolution of the transfer curves (drain current versus gate voltage) as a function of light illumination time. The a-IGZO TFTs show very good electrical properties with mobility above 10 cm^2^ V^−1^ s^−1^ and on/off ratio of ~10^8^ under darkness condition. Upon the exposure of the a-IGZO TFTs to light illumination, the V_th_ is negatively shifted (>10 V). In contrast, the a-IGZO TFTs passivated by the Si-Ge photo blocking layers show no change in electrical properties during the light illumination, as shown in Fig. 4d. This result suggests a superior possibility of the Si-Ge films as a photo blocking layers for the a-IGZO based industrial display field under light illumination.

**Figure 5:**
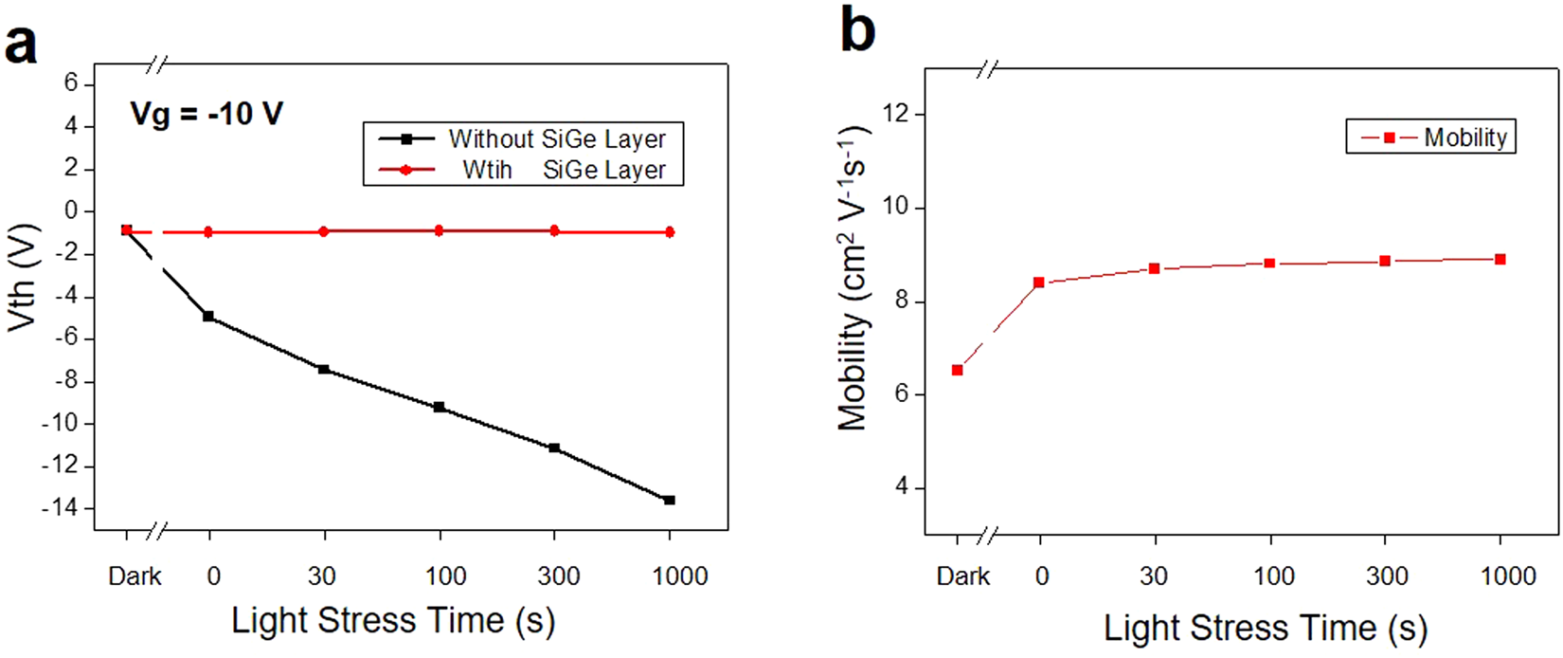
Vth and mobility of a-IGZO TFTs passivated by the Si-Ge films depending on the light stress time. (**a**) Vth change of a-IGZO TFTs passivated by the Si-Ge photo-blocking layers as a function of the light illumination compared with pristine a-IGZO. (**b**) Electron mobility change of a-IGZO TFTs passivated by the Si-Ge photo-blocking layers depending on light stress time.

In order to obtain more information of electrical properties on the a-IGZO TFTs passivated by the Si-Ge photo-blocking layers, the effect of light stress time on the change of V_th_ and electron mobility was investigated in detail. The V_th_ changes without and with the Si-Ge passivation layer to the light illumination condition are compared, as shown in Fig. 5a. By applying 10 V gate voltage, V_th_ of pristine a-IGZO is largely negatively shifted (~15 V at 1000 sec) in the direction with increasing the light stress time, while that of the Si-Ge passivated a-IGZO persists similar value compared with initial value. Furthermore, the electron mobility of the a-IGZO TFTs passivated by the Si-Ge layers shows no change under the light stress time, as shown in Fig. 5b. Therefore, the V_th_ shift and electron mobility are greatly dependent on the existence of the Si-Ge photo-blocking layers.

## Discussion

In conclusion, we synthesized the Si-Ge films by PECVD and investigated the effects of the photo blocking Si-Ge layers on the electrical properties of a-IGZO semiconductors. Although the thicker Si-Ge films shows stronger the light absorbance, the electrical performance of a-IGZO TFTs is inversely proportional to the thickness of the Si-Ge films because of accumulated free electrons. The thickness of the dielectric buffer layers changes the charge capacitance and surface instability of the TFTs, which also considerably affects the device performance. Therefore, we optimize the thicknesses of the Si-Ge films (~200 nm) and the buffer layers (~300 nm). After up to 1,000 s exposure to light, the V_th_ and electron mobility of the a-IGZO TFTs passivated by the Si-Ge photo-blocking layers were unchanged. Considering the simple fabrication process by PECVD with outstanding scalability, we expect that this method can be widely applied to various metal-oxide TFT devices that are sensitive to light illumination.

## Methods

### Synthesis

Using plasma enhanced chemical vapour deposition (PECVD), the 100 nm SiO_2_ layers were deposited on a glass substrate for the efficient synthesis of the Si-Ge films. The SiO_2_/glass substrate was placed on a quartz flat inside of 4-inch quartz tube. The Si-Ge films were synthesized on SiO_2_/glass substrates through the PECVD method, using silane (10 sccm) and germane (10 sccm) with vacuum pumping at 370 °C at initial low vacuum (~25 mTorr). After 5~10 min of direct exposure to the plasma (100 W) at that temperature, a large-area Si-Ge films formed on the SiO_2_/glass substrate. Then, silicon nitride buffer layers also deposited on the substrate using PECVD with same method above.

### Characterization

The entire structure was investigated by field-emission scanning electron microscopy (FESEM, AURIGA Carl Zeiss). The crystal phases were collected using an X-ray diffractometer (D8-Advance, Bruker Miller Co.) with Cu Ka1 irradiation. The Raman spectra were obtained by a Raman spectrometer (RM 1000-Invia, Renishaw, 514 nm). Structure bonding in the structure is analysed by XPS carried out with a KRATOS AXIS-His model in Research Institute of Advanced materials. The electrical properties were measured by Agilent 2602.

## Electronic supplementary material

Supplementary Information

## References

[1] Kim Minkyu, Jeong Jong Han, Lee Hun Jung, Ahn Tae Kyung, Shin Hyun Soo, Park Jin-Seong, Jeong Jae Kyeong, Mo Yeon-Gon, Kim Hye Dong (2007). High mobility bottom gate InGaZnO thin film transistors with SiOx etch stopper. Applied Physics Letters.

[2] Fortunato EMC (2005). Fully transparent ZnO thin-film transistor produced at room temperature. Adv. Mater..

[3] Ohta H (2004). Transparent oxide optoelectronics. Mater. Today.

[4] Xiaochen M (2017). A Sputtered Silicon Oxide Electrolyte for High-Performance Thin-Film Transistors. Sci. Reports.

[5] Nomura KH (2004). Room-temperature fabrication of transparent flexible thin-film transistors using amorphous oxide semiconductors. Nature.

[6] Hirao T (2007). Novel top-gate zinc oxide thin-film transistors (ZnO TFTs) for AMLCDs. J. Soc. Inf. Display.

[7] Park SHK (2009). Transparent and photo-stable ZnO thin-film transistors to drive an active matrix organic-light-emitting diode display panel. Adv. Mater..

[8] Kwon JY (2008). Bottom-gate gallium indium zinc oxide thin-film transistor array for high-resolution AMOLED display. IEEE. Elec. Dev. Lett..

[9] Li X (2014). High-speed dual-gate a-IGZO TFT-based circuits with top-gate offset structure. IEEE. Elec. Dev. Lett..

[10] Ji KH (2010). Comparative study on light-induced bias stress instability of IGZO transistors with SiNx and SiO2 gate dielectrics. IEEE. Elec. Dev. Lett..

[11] Park Sang-Hee Ko, Ryu Minki, Yoon Sung Min, Yang Shinhyuk, Hwang Chi-Sun, Jeon Jae-Hong, Kim Kyounghwan (2011). Light Response of Top Gate InGaZnO Thin Film Transistor. Japanese Journal of Applied Physics.

[12] Chen WT (2012). High-performance light-erasable memory and real-time ultraviolet detector based on unannealed indium-gallium-zinc-oxide thin-film transistor. IEEE. Elec. Dev. Lett..

[13] Jiang D. L., Li L., Chen H. Y., Gao H., Qiao Q., Xu Z. K., Jiao S. J. (2015). Realization of unbiased photoresponse in amorphous InGaZnO ultraviolet detector via a hole-trapping process. Applied Physics Letters.

[14] Mativenga M (2012). Performance of 5-nm a-IGZO TFTs with various channel lengths and an etch stopper manufactured by back UV exposure. IEEE. Elec. Dev. Lett..

[15] Gosain Dharam Pal, Tanaka Tsutomu (2009). Instability of Amorphous Indium Gallium Zinc Oxide Thin Film Transistors under Light Illumination. Japanese Journal of Applied Physics.

[16] Kiazadeh Asal, Gomes Henrique L., Barquinha Pedro, Martins Jorge, Rovisco Ana, Pinto Joana V., Martins Rodrigo, Fortunato Elvira (2016). Improving positive and negative bias illumination stress stability in parylene passivated IGZO transistors. Applied Physics Letters.

[17] Bak JY (2014). Impact of charge-trap layer conductivity control on device performances of top-gate memory thin-film transistors using IGZO channel and ZnO charge-trap layer. IEEE. Elec. Dev. Lett..

[18] Ok KC (2015). Effect of alumina buffers on the stability of top-gate amorphous InGaZnO thin-film transistors on flexible substrates. Oh, H. J. IEEE. Elec. Dev. Lett..

[19] Liu PT (2011). Ambient stability enhancement of thin-films transistor with InGaZnO capped with InGaZnO:N bilayer stack channel layers. IEEE. Elec. Dev. Lett.

[20] Sundholm ES (2012). Passivation of amorphous oxide semiconductors utilizing a zinc-tin-silicon-oxide barrier layer. IEEE. Elec. Dev. Lett..

[21] Humlicek J (1989). Optical-spectra of SixGe1-x alloys. J. Appl. Phys..

[22] Luo, W. *et al*. Efficient fabrication of nanoporous Si and Si/Ge enabled by a heat scavenger in magnesiothermic reactions. *Sci*. *Reports* **3** (2013). 10.1038/srep02222 PMC3713525 23860418

[23] Alonso MI (1989). Raman-spectra of C-Si1-xGex alloys. Phys. Rev. B.

[24] Chen YC (2013). Dependence of light-accelerated instability on bias and environment in amorphous indium-gallium-zinc-oxide thin film transistors. ECS J. Solid State Sci..

[25] Goodman NB (1980). Analysis of field-effect and capacitance-voltage measurements in amorphous-semiconductors. Philos. Mag..

[26] Alshammari FH (2016). Enhanced ZnO thin-film transistor performance using bilayer gate dielectrics. ACS Appl. Mater. Inter..

