# Supplementary material for: Silicon germanium photo-blocking layers for a-IGZO based industrial display
Source: Sci Rep. 2018 Dec 3;8:17533. doi: 10.1038/s41598-018-35222-9 (PMC6277433; doi:10.1038/s41598-018-35222-9)
Supplement: Supplementary file 1 — Supplementary Information [file 41598_2018_35222_MOESM1_ESM.docx]

Supporting Information

Silicon germanium photo-blocking layers for a-IGZO based industrial display

Su Hyoung Kang^1,†^, Sangmin Kang^1,2,†^, Seong Chae Park^3^, Jong Bo Park^1^, Youngjin Jung^1^ and Byung Hee Hong^1,3,*^

^1^ Department of Chemistry, College of Natural Science, Seoul National University, Seoul 440-746, Korea

^2^ Department of Electrical and Computer Engineering, University of Illinois at Urbana-Champaign, Urbana, Illinois 61801, USA

^3^ Graduate School of Convergence Science and Technology, Seoul National University, Suwon 433-270, Korea

^†^ These authors contributed equally to this work.

* Correspondence and requests for materials should be addressed to B. H. H. (email: byunghee@snu.ac.kr)


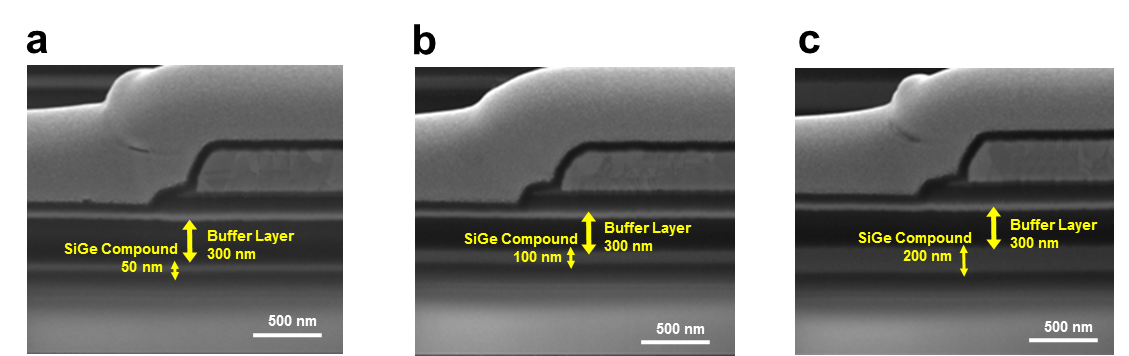


**Figure S1. Side view SEM images of a-IGZO TFT at dielectric buffer layers 300 nm.** (a) The Si-Ge films 50 nm TFTs. (b) The Si-Ge films 100 nm TFTs. (c) The Si-Ge films 200 nm TFTs.


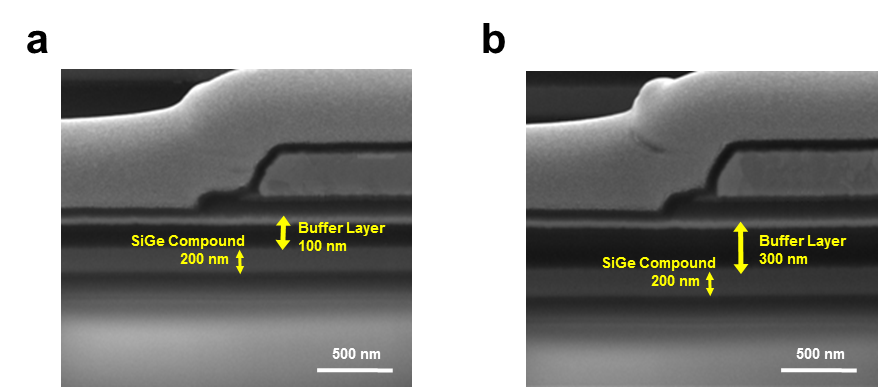


**Figure S2. Side view SEM images of a-IGZO TFT at the Si-Ge films 200 nm.** (a) The dielectric buffer layers 100 nm. (b) The dielectric buffer layers 300 nm.
